# Evaluating drug use patterns among paediatric outpatients in Burundi

**DOI:** 10.1080/20523211.2024.2312369

**Published:** 2024-02-29

**Authors:** Audace Manirakiza, David Gitonga Nyamu, Shital Mahindra Maru, Thomas Bizimana, Manassé Nimpagaritse

**Affiliations:** a Master of Health Supply Chain Management at the East African Community Regional Centre of Excellence for Vaccines, Immunization, and Health Supply Chain Management, College of Medicine and Health Sciences, University of Rwanda, Kigali, Rwanda; b Department of Pharmacology, Clinical Pharmacy and Pharmacy Practice, Faculty of Health Sciences, University of Naïrobi, Nairobi, Kenya; c Faculty of Health Sciences, Department of Pharmaceutical Chemistry, Pharmaceutics and Pharmacognosy, University of Naïrobi, Nairobi, Kenya; d Department of Research, Institut National de Santé Publique, Bujumbura, Burundi

**Keywords:** Rational medicine use, primary healthcare centres, prescribing indicators, specific-facility indicators, Bujumbura Mairie, Burundi

## Abstract

**Background:**

Rational prescribing is key to optimising therapeutic outcomes and avoiding risks associated with irrational use of medicines. Using WHO drug use indicators, this study evaluated drug use patterns among paediatric outpatient encounters at Primary Healthcare Centers (PHCs) in Bujumbura Mairie, Republic of Burundi.

**Methods:**

Descriptive cross-sectional research assessed paediatric medicine use in 20 PHCs. From 8 February to 7 April 2023, 800 randomly selected paediatric encounters’ 2022-year data were retrospectively collected. Data for specific facility indicators were prospectively collected. SPSS 23 was used to analyse data.

**Results:**

800 outpatient child encounters were analysed, 48.4% female and 51.6% male. The mean number of medicines per encounter was 2.4(±0.99). The injection rate was 9.9%. Overall, 78.8% of generics and 85.2% of essential medicines were prescribed. Results show drug prescribing differences between private and government PHCs (*p *< 0.001). All PHCs studied had no standard treatment guidelines (STGs), while 50% had an essential medicine list (EML) and 85% of key medicines were available.

**Conclusion:**

Poor prescribing practices were found indicating the need for interventions to promote good drug use practices. A large study at a national scale is required to provide a more comprehensive understanding of the overall drug use practices.

## Background

Rational medicine use is key in improving patient and community health (MSH, 2012; USAID, 1985; WHO, 2002). It is defined by the World Health Organization (WHO) as patients getting the right medications for their clinical needs, at the right doses, for the right amount of time, and for the least amount of money for them and their community (WHO, 2002; 1987). According to WHO, in developing nations, more than half of the medicines that are prescribed to patients, dispensed by health professionals, and sold either in healthcare facilities or in retail pharmacies are done so in an inappropriate manner (Hogerzeil, 1995; WHO, 2002). Irrational prescribing is one of the most significant challenges facing healthcare systems worldwide (Ofori-Asenso & Agyeman, 2016).

Moreover, this results in treatment failure (WHO, 2002). Irrational prescribing not only leads to misuse of the drugs but also contributes to an increase in the costs associated with medical care, patient impairment as a result of prolonged hospitalisation, and treatment failure (Atif et al., 2016a; Bell et al., 2014; Klein et al., 2021; Romandini et al., 2021).

Drug use indicator studies have been successfully conducted in developing countries including Africans. For instance, a study conducted in four hospitals in Yemen, in 2015 revealed irrational prescribing patterns at outpatient departments (Alshakka et al., 2016). In addition, a study conducted in 2010 in Alexandria (Egypt) showed irrational drug use at primary healthcare centres (Akl et al., 2014). Moreover, the results of a systematic review of studies conducted from 1995 to 2015 in Africa using prescription drug indicators significantly deviated from the World Health Organization's optimal values. These findings highlight the need for improved medicine prescribing and access to appropriate healthcare in Africa (Ofori-Asenso et al., 2016).

The studies carried out in East African Countries revealed non-implementation of the core drug use indicators as recommended by WHO. In Rwanda, a study carried out in child prescribing indicators in 2017 revealed a big gap in implementing WHO indicators (Habimana et al., 2020). According to a drug prescribing evaluation conducted in four Tanzanian regions in 2014 revealed injectable and antibiotic irrational prescribing (Irunde et al., 2017). In addition, a 2018 drug use indicators study conducted in Kisii county, Kenya, revealed irrational medicines use, indicating the need for interventions to address issues (Nyabuti et al., 2020). In many developing nations, children account around 40% of the total population (Kumar et al., 2020) and studies on drug use revealed a higher rate of irrational medicines prescribing among paediatrics (Abu Farha et al., 2022; Rahul et al., 2021; Thomas et al., 2015). However, in the republic of Burundi, data on paediatric drug use is scarce. Despite the existence of Drug Therapeutic Committee and remarkable improvement in the last two decades in Burundi, it is important to note that the current National Medicine Policy (NMP) is outdated and not disseminated, while the STG is now non-existent. In domain of rational medicine use, the National Medicine Policy holds the highest level of disseminated policy.

For proper drug prescribing, research to determine the scope of irrational drug use are necessary in order to develop and put into practice corrective measures that will encourage rational prescribing (Galappatthy et al., 2021b). In this way, the existing healthcare system would be able to guarantee that patients receive treatments that are both safe and effective.

In order to achieve this objective, the World Health Organization (WHO) and the International Network for the Rational Use of Drugs (INRUD) have developed indicators or basic parameters that enable researchers to evaluate the use of drugs in healthcare facilities. These metrics comprise prescribing, specific facility, and patient-care indicators (Holloway & Green, 2003; WHO, 1993).

The objective of this study was to evaluate drug use patterns among paediatric outpatient encounters at Primary healthcare Centers (PHCs) in Bujumbura Mairie, Republic of Burundi using WHO/INRUD prescribing and specific-facility core indicators.

## Methods

### Study design and population

A descriptive cross-sectional study was conducted to assess medicine use pattern for paediatrics in 20 PHCs in Bujumbura Mairie, Republic of Burundi. The research was a restrospective and prospective study respectively for prescribing and specific-facility indicators. Data collection was conducted from 8 February to 7 April 2023. Reffering to WHO guidelines, core prescribing and facility indicators data was collected. Prescribing data was collected from medical records information among paediatric population (<18 years) consultations during 2022-year-period while data for specific facility indicators were collected prospectively. In Burundi, according to 2021 health statistics yearbook, 22% of younger-than-24-year-old patients were seen as outpatients in the healthcare facilities.

### Study instruments

After a comprehensive literature review of similar studies, data collection forms for this study were drawn from WHO/INRUD validated forms (Akl et al., 2014; Amaha et al., 2019; Kilipamwambu et al., 2021). The researcher adhered to the WHO/INRUD guidelines and utilised the WHO standard prescribing and facility-specific indicator forms designed for this purpose (Holloway & Green, 2003; WHO, 1993).

### Sample size

According to WHO guidelines, a minimum sample size of 600 encounters is recommended to assess drug use indicators (Holloway & Green, 2003; WHO, 1993). To accomplish this, randomly selected using excel formula, 800 studied paediatric outpatient encounters were obtained from the 20 PHCs by retaining only paediatric data from the 100 encounters all ages considered per each PHC and distributed at regular intervals among each PHC’s total encounters in 2022-year period. In addition, according to WHO recommendations, a max of 100 prescriptions should be obtained at one facility to investigate drug use, and then a sample of 20 facilities, drawn from a same type of health facilities within a single region, should be sampled for greater reliability (Holloway & Green, 2003; WHO, 1993). Therefore, the 20 PHCs were selected randomly from the PHCs of Bujumbura Mairie health province using an Excel formula after ranking them alphabetically.

### Variable measurement

To calculate indicators, the formula approved by WHO’s guidelines to evaluate drugs use were used as follows:

1. Prescribing indicators evaluation:

- Average drugs number per encounter

Calculated by dividing the total number of drugs prescribed by the total number of encounters evaluated.

- Percentage of drugs prescribed by generic name

Calculated by dividing the total number of generic medicines prescribed by the total number of medicines prescribed and then multiply by 100 to make percentage.

- Percentage of encounters with at least an injection prescribed

Divide the total number of patients who received one or more injections by the total number of encounters and multiply by 100 to make a percentage.

- Percentage of drugs prescribed from EML

Divide the total number of EML drugs prescribed by the total number of drugs prescribed and multiply by 100 to make a percentage.

2. Health facility specific evaluation:
  - Availability of copy of EML

This indicator reads either yes or no, for the facility as a whole.

- Availability of Standard Treatment Guidelines (STG)

This indicator reads either yes or no, for the facility as a whole.

- Availability of key drugs

A column for the number of key drugs in stock was added and then divided by the total number of key drugs surveyed and multiply by 100 to get a percentage. This should be expressed without decimals.

In addition, to assess drug usage comprehensively, Indices such as Index of Rational Facility Specific Drug Use (IRFSDU) and Index of Rational Drug Prescribing (IRDP) were developed by Zhi and Zhang (Akl et al., 2014; Siele et al., 2022; Wendie et al., 2021). To calculate indices of non-pharmacy and injection use safely, the following formula was applied.

$$\mathrm{Index}=\frac{\mathrm{Optimal}\;\mathrm{value}\;(\mathrm{WHO}\;\mathrm{standard})}{\mathrm{Observed}\;\mathrm{value}}$$

The recommended values by the WHO for calculating non-polypharmacy and injection-safe use indices were 1.8 and 24.1, respectively.

The following formula was used to calculate other indices, including the indexes for generic prescribing, EML prescribing, EML copy availability, and key drug availability:

$$\mathrm{Index}=\frac{\mathrm{Observed}\;\mathrm{value}}{\mathrm{Optimal}\;\mathrm{value}\;(\mathrm{WHO}\;\mathrm{standard})}$$

For all these other indices, the WHO optimal values were set at 100. The closer the calculated index value is to 1, the more the rational drug usage indicator.

### Statistical analysis

Raw data entry was done in Microsoft Excel version 2022 and exported to Statistical Package for the Social Sciences (SPSS) version 23 for analysis. In descriptive statistical analysis, continuous variables were analysed by using the mean, IQR, and standard deviation, while for categorical variables, frequencies and percentages were used. Mann–Whitney U and Kruskal–Wallis tests were utilised to demonstrate the variation in prescribing drugs based on demographics.

Trends in medicine prescribing and specific facility indicators were computed, evaluated, and compared to WHO standard values (Amaha et al., 2019; Atif et al., 2016b).

## Results

### Socio-demographic characteristics of the outpatient children

Table 1 shows the social demographic characteristics of the outpatient children studied. The median age of children was 5.0 years (IQR = 10) and 413 (51.6%) of the participants were male while female accounted for 387 (48.4%). Patients were principally visiting the private healthcare facilities (666, 83.3%) as opposed to public medical centres (134, 16.8%).

**Table 1.** Socio-demographic characteristics of the outpatient children (*n* = 800).

Variables	Frequency	Percent
Age category (Years)		
< 5	342	42.8
5–14	336	42.0
15–17	122	15.2
Gender		
Male	413	51.6
Female	387	48.4
Healthcare category		
Private	666	83.2
Government owner	134	16.8

### Prescribing indicators

The pattern of the prescribing indicators is shown in Table 2. There were 800 patients analysed prescribed 1882 medicines giving an average of 2.4 (±0.99) drugs per encounter. The total number of generic drugs prescribed was 1483 (78.8%), while the total number of EML drugs prescribed was 1603 (85.2%). The total number of encounters in which at least one injection was prescribed was 79 (9.9%).

**Table 2.** Pattern of prescribing indicators among outpatient children.

Prescribing indicators	Total medicines or encounters (frequencies)	% (±SD if applicable)	Average (±SD)	WHO optimal values
Average number of medicines prescribed per patient encounter	1882	–	2.4 (±0.99)	1.6–1.8
% drugs prescribed by generic name	1483	78.8(±1.09)	–	100
% encounters with an injection prescribed	79	9.9	–	13.4–24.1
% drugs prescribed from EML	1603	85.2(±1.03)	–	100

Key: EML: essential medicines list.

It is important to note that 2 of the paediatric outpatients received three injectables in their prescriptions, 11 received two injectables while 66 received one. In addition, the types and frequencies of prescribed injectables among the study sample are as the following: metamizole (64 times), hydrocortisone (5 times), diclofenac (5 times), ceftriaxone (3 times), cefotaxime (3 times), dexamethasone (3 times), metoclopramide (3 times), aminophylline (2 times), gentamicin (2 times), hyoscine (2 times), cimetidine (1 time), and phloroglucinol/trimethyl phloroglucinol (1 time).

Table 3 demonstrates the variation in prescribing drugs after testing the normality distribution of drugs among participants regarding sociodemographic characteristics. Since the *p*-value for normality test is significant (*p *< 0.001), the data were not normally distributed. This allowed to perform Mann Whitney U Test and Krustal Wallis Anova. Results obtained showed that there is a difference between Private and government owner PHCs in prescribing drugs.

**Table 3.** Difference in prescribing drugs based on socio-demographics.

Socio-demographics	Number of drugs			
	Normality test		Other tests	
Variables	Kolmogorov Smirnov (*p-*value)	Shapiro-Wilk (*p-*value)	Mann Whitney U Test (*p-*value)	Kruskal Wallis Anova (*p-*value)
Age category (Years)				0.938
< 5 (*n* = 342)	<0.001	<0.001		
5–14 (*n* = 336)	<0.001	<0.001		
15–17 (*n* = 122)	<0.001	<0.001		
Gender			0.641	
Male (*n* = 413)	<0.001	<0.001		
Female (*n* = 387)	<0.001	<0.001		
Healthcare centre category			<0.001	
Private (*n* = 666)	<0.001	<0.001		
Government owner (*n* = 134)	<0.001	<0.001		

### Specific facility indicators

The findings for facility-specific indicators revealed that the availability of a copy of EML was 50%, and key medicines were at 85%. STGs were not available in all surveyed facilities. The optimal values should be at 100% of availability for each indicator.

### Performance indicators for selected primary healthcare facilities

Table 4 below shows the indices of performance for specific facility and prescribing indicators. The indexes for key medicine availability, and a copy of EML availability were 0.85, and 0.50, respectively. For medicine prescribing indicators, only the index of injection prescription reached the optimal value of 1.

**Table 4.** Indices of performance for specific facility and prescribing indicators.

Performance indicators	Performance index	Optimal index
Medicine prescribing indicators		
Non-polypharmacy index	0.75	1
Generic name index	0.78	1
Essential medicine list index	0.85	1
Rational injection safety index	1	1
*IRDP*	*3.38*	*4*
Facility specific indicators		
% availability of key medicines	0.85	1
% availability of a copy of EML	0.50	1
*IRFSDU*	*1*.*35*	*2*

Key: IRDP: index of rational drug prescribing; IRFSDU: index of rational facility-specific drug use.

## Discussion

The present research revealed drug use patterns in public and private healthcare centres of Bujumbura Mairie, Republic of Burundi. Except for injectables, other prescribing findings related to average drug number in a prescription, generic, and essential medicines prescribing did not fall within the optimal values set by the WHO. In addition, this research revealed that all facility-specific indicators were suboptimal compared to the ones recommended by the WHO.

### Prescribing indicators

The findings from the present study on average number of medicines per encounter were compared to results from prescribing indicator studies conducted in Ethiopia (1.95), Nigeria (3.4), Tanzania (1.99), Sudan (2.4), Ghana (3.5), Nepal(2.6), India (6.12), Sri Lanka (3.1), Sierra Leone (3.6), China(2), Iran (3.14), Brazil (2.4), and 3.1 for African region (1995–2015 systematic analysis) (Abdin & Mohammed, 2021; Ahmadi & Zarei, 2017; Aryal et al., 2020; Galappatthy et al., 2021b; Jin et al., 2019; Kabba et al., 2020; Kilipamwambu et al., 2021; Lima et al., 2017; Mathew et al., 2021; Ofori-Asenso et al., 2016; Okoye et al., 2022; Prah et al., 2017; Yimer et al., 2022).

This study found that the average number of medicines prescribed per encounter was 2.4(±0.99), exceeding both the WHO-recommended value (1.6–1.8) and African standards (<2) (Amaha et al., 2019; Atif et al., 2016b). This finding was the same in Sudan (2.4) and Brazil (2.4) (Abdin & Mohammed, 2021; Lima et al., 2017). Nonetheless, this was greater than the figures reported for Ethiopia (1.95), Tanzania (1.99), and China (2) (Jin et al., 2019; Kilipamwambu et al., 2021; Yimer et al., 2022) which also exceeded the WHO-recommended values.

Noticeably higher findings were found from the studies carried out in Nigeria (3.4), Ghana (3.5), Nepal (2.6), India (6.12), Sri Lanka (3.1), Sierra Leone (3.6), Iran (3.14), and African region systematic analysis (3.1).

According to this research, the higher number of drugs prescribed per encounter could be attributed to a lack of prescribing guidelines (STGs) or regular therapeutic training, and practices influenced by patient demand.

Therefore, measures should be put in place to educate and increase prescribers’ awareness on the value of rational drug use, and how it affects patient therapeutic adherence, adverse medicine events, and interactions of drugs.

Since the average number of drugs prescribed per encounter was exceeding the optimal value, it was crucial to detect the variation in prescribing drugs among socio-demographic characteristics. The results showed variation between private- and government owned PHCs (*p*-value = 0.000). Therefore, further studies are required to demonstrate which PHC category prescribe more drugs and ascertain the causes of this trend.

The study revealed that 78.9% of the medicines were prescribed by generic names. These findings were higher than the WHO optimal value (100%). Comparing these results to others from previous similar studies, lower results were found in India (15.96%), Nepal (57.7%), United Arab Emirates (UAE) (58.6%), Pakistan (56.6%), Sri Lanka (35.5%), Kenya (27.7%), Ghana (76%), Sudan (49.8%), and African region systematic review (68%) (Abdin & Mohammed, 2021; Amponsah et al., 2022; Atal et al., 2021; Atif et al., 2016b; Galappatthy et al., 2021b; Nyabuti et al., 2020; Ofori-Asenso et al., 2016; Rabbani et al., 2023; Shrestha et al., 2021). These lower results could be attributed to the non-establishment or non-enforcement of the regulatory policies. In addition, it could also be due to promotion of branded drugs.

In addition, studies conducted in Northwest Ethiopia (94%), Iran (95.1%), and China (97%) found higher values than those of the current study (78.9%) (Ahmadi & Zarei, 2017; Jin et al., 2019; Sema et al., 2021).

Therefore, efforts should be made to promote the use of generics instead of branded medicines by implementing policies to reduce costs and increase affordable drug accessibility.

The percentage of encounters in which a patient was prescribed at least one injection in the present study was 9.9%, which is closer the value found in 2014 Burundi outpatient encounter study all ages considered (Holloway & Henry, 2014), was lower than the value recommended by WHO (13.4–24.1). Results were similar to those from studies conducted in India (1.98%; 0.64%), Northwest Ethiopia (9.5%), Nepal (0.6%, 4.2%), UAE (9.5%; 0.5%), Pakistan (0%), Sri Lanka (1.2%), Ghana (7%), and in Rwanda (1.2%) (Abu Farha et al., 2022; Amponsah et al., 2022; Aryal et al., 2020; Atal et al., 2021; Atif et al., 2016b; Galappatthy et al., 2021b; Habimana et al., 2020; Mandal et al., 2022; Rabbani et al., 2023; Sema et al., 2021; Shrestha et al., 2021). In contrast, the results that exceeded the WHO optimal values were revealed from Iran (24.4%), the 1995–2015 African region systematic review (25%), and Kenya (24.9%) (Ahmadi & Zarei, 2017; Nyabuti et al., 2020; Ofori-Asenso et al., 2016).

Even though the percentage of encounters in which a patient was prescribed at least one injection in the present study was lower than the value recommended by WHO, the most prescribed injectable, metamizole, was not found in the EML of Burundi. In addition, as per of Burundi EML, ceftriaxone, cefotaxime, dexamethasone, metoclopramide, aminophylline, cimetidine, and phloroglucinol/trimethyl phloroglucinol prescribed were excluded from PHC injectables (MSPLS, 2022).

The higher cost value of injectables compared to other forms of medication may also contribute to their limited utilisation. Additionally, injectables are most commonly used for inpatients (Habimana et al., 2020).

Reducing the use of injectables is a positive measure, especially since the high use of injectable drugs necessitates the use of invasive devices, which increases the risk of contracting some communicable diseases such as HIV/AIDS and other blood-borne infectious due to the utilisation of equipment which are not sterile (Osório et al., 2023). It is important to prioritise strategies for continuing reducing injectable drug use to mitigate the potential risks.

This study revealed that 85.2% of medicines were prescribed from the EML, which is lower than the WHO-recommended value (100%). However, the 100% rate had not been reached maybe due to an insufficient supply of EML drugs. The finding is significantly less than the results from similar studies conducted in Tanzania (97.6%), Eritrea (98.3%), Pakistan (93.4%), Ethiopia (100%), Kenya (96.7%), Nigeria (89.6%), and Iran (95.9%) (Ahmadi & Zarei, 2017; Amaha et al., 2019; Atif et al., 2016a; Kilipamwambu et al., 2021; Nyabuti et al., 2020; Okoye et al., 2022; Sema et al., 2021; Yimer et al., 2022).

Even though the percentage of drugs prescribed from EMLs were lower compared to WHO optimal value for the current study, the results were higher than more other findings revealed from studies conducted in India (37.37%; 58.3%), Nepal (65.8%; 80.9%), UAE(47.7%; 83.8%), Sri Lanka (68.8%), and Sudan (81.19%) (Abu Farha et al., 2022; Aryal et al., 2020; Atal et al., 2021; Galappatthy et al., 2021b; Mandal et al., 2022; Rabbani et al., 2023; Shrestha et al., 2021; Yousif & Supakankunti, 2016).

### Specific facility indicators

The current study revealed that 50% of PHCs lacked a copy of EML, none of them had a copy of STGs, and the key drugs were available at a rate of 85%. These findings were lower than the WHO optimal values for EMLs, STGs, and key drug availability set to 100%.

The results for EML copies availability for the current study (50%) were found lower compared to the findings revealed from similar studies conducted in Northwest Ethiopia (100%), in Pakistan (100%), Nigeria (98.8%) and Nepal (83.3%) (Adeosun et al., 2022; Aryal et al., 2020; Atif et al., 2016b; Sema et al., 2021), however, higher than results found from Northeast Ethiopia study (0%) (Wendie et al., 2021). Rational prescribing uses EML drugs to provide affordable, effective, and safe medicines. The EML is a carefully selected list of drugs for the most common health issues in a population. Prescribers can improve patient outcomes, reduce drug use, and lower costs by following the EML. Since some healthcare facilities did not have copies of the EML, prescribers could prescribe medicines that are not on the EML (Atif et al., 2016a).

In addition, the current findings for STGs availability (0%) were lower compared to the findings revealed from similar studies conducted in Northwest Ethiopia (100%) (Sema et al., 2021). The purpose of developing and implement STGs is to ensure that patients receive the most appropriate and evidence-based care for their specific conditions. However, without STGs, prescribers may prescribe less effective or inappropriate drugs for common health conditions of a specific health system. This can cause poor therapy and impair patient outcomes.

Moreover, this study revealed that Key drug availability was at the rate of 85%, which was higher compared to the findings from studies conducted in Northwest Ethiopia (83.5%), Northeast Ethiopia (64.1%), Pakistan (72.4%), and Nepal (64.7%) (Aryal et al., 2020; Atif et al., 2016b; Sema et al., 2021; Wendie et al., 2021), but lower than results found from Nigeria study (89.8%) (Adeosun et al., 2022).

These results suggest that there is a significant gap in the implementation of essential medicines policies at primary healthcare centres. The lack of STGs combined with low rate of EML policy availability may contribute to suboptimal prescribing practices and potentially compromise patient care. The pharmaceutical industry can benefit from the development and subsequent implementation of STGs and the enforcement of EML policy by aligning with international prescribing standards.

### Indexes of performance indicators

This study discovered that the four-IRDP was 3.38, which is less than the optimal four. In addition, when evaluating the four indicators, this result was lower than the 3.89 and 3.83 revealed, respectively, by the Eritrea regional and national referral hospitals study, North-East Ethiopia (Siele et al., 2022; Wendie et al., 2021). However, this is higher than the 2.78 reported from Sri Lanka study (Galappatthy et al., 2021a).

This study revealed that the IRFSDU was 1.35 out of 2.0, which was lower than the findings from Alexandria in Egypt (1.75), Eritrea (1.801), but higher than the findings from a review of Ethiopian studies (1.01) (Akl et al., 2014; Mohammed & Faris, 2021; Siele et al., 2022).

These findings highlight the necessity for policymakers to implement interventions as a prompt solution for rational drug use.

## Limitation of the study

In general, this study addressed WHO prescribing and specific-facility indicators in selected PHCs. In fact, it highlighted the most significant problems and quantified their magnitude. They do not explain, however, why the problems exist. Moreover, these indicators did not reveal whether the prescribed medications are appropriate for the diagnosis. Moreover, the findings cannot be generalised to the whole country as only a few PHCs were involved in. Furthermore, the prescribing patterns for inpatients were also not evaluated. Also, the underlying reasons or factors influencing prescribing were not explored.

## Conclusion

The present study revealed irrational prescribing patterns among paediatric outpatient encounters in Bujumbura Mairie. Except for the number of injections, the results in the terms of specific facility and prescribing indicators were divergent with the WHO standards.

In addition, the results highlighted a need for implementing stricter regulations and monitoring systems to ensure adherence to the WHO guidelines. Therefore, collaborations between healthcare professionals and policymakers should be established to develop policies and strategies that promote rational drug use and improve prescribing practices across various healthcare facilities. Moreover, further national research is needed to understand medication use at a national scale.

## Data Availability

Dataset is available and can be shared.

## Abbreviations

Abbreviations: EML: essential medicines list; NMP: National Medicine Policy; INRUD: International Network for the rational use of drugs; IRDP: index of rational drug prescribing; IRFSDU: index of rational facility specific drug use; IQR: inter-quartile range; WHO: World Health Organization; STG: standard treatment guidelines; UAE: United Arab Emirates; SPSS: Statistical Package for the Social Sciences.
